# Page for the General Public

**DOI:** 10.12945/j.aorta.2017.17.097

**Published:** 2018-09-24

**Authors:** Anneke Damberg, on behalf of the Editorial Office

The following pages summarize and review this issue’s articles for an audience without a background in medicine or research.

## Original Research Articles

*Diken et al.: “Distribution of calcifications of thoracic aorta in patients undergoing coronary artery bypass grafting“*

 When patients need coronary artery bypass surgery, the vessels affected by coronary disease, [which impairs blood flow to the heart vessel,] are bypassed by graft vessels. In many cases, manipulation of the aorta, the body’s main artery, is necessary to attach grafts or insert tubes during the procedure. If the aorta is calcified, calcifications can come loose during manipulation and cause e.g. a stroke by occluding vessels in the brain. Analysing imaging of 443 patients, *Diken et al.* studied the distribution of calcifications in the aorta which were present in 67% of patients. They proposed a classification which can be used for patients undergoing coronary artery bypass surgery.

*De Martino et al.: “Surgical treatment of annuloaortic ectasia - Replace or repair?“*

 When the body’s main artery, the aorta, is enlarged at its root, it often causes one of the heart’s valves, the aortic valve, to leak. When surgery is necessary, this valve can either be replaced or repaired. *De Martino et al.* studied the results of 181 patients who underwent either of these procedures. They came to the conclusion that both are associated with good outcome. Among patients with aortic valve replacement, bleeding and strokes were more common. The authors suggest to save the aortic valve whenever possible. However, this study has a limited design and relevant differences in follow-up between the two patient groups existed. The results therefore need validation in further studies. 

*Jasinski et al.: “Factors affecting follow-up compliance in patients following endovascular aneurysm repair“*

 After undergoing an intervention for an aortic aneurysm, a dilatation of the body’s main artery, lifelong follow-up imaging is important to recognize complications or progress of the disease. However, many patients fail to show up for follow-up tests. *Jasinski et al.* analysed the data of 205 patients who underwent endovascular aortic procedures, a procedure in which a graft prosthesis is inserted into the aorta via a smaller vessel, e.g. in the groin. They noted that about 50% of patients failed to come to follow-up appointments. Especially patients who underwent emergency procedures, widowed patients, minority patients and patients who live far away from the outpatient clinic did not come. The authors implemented a follow-up system to remind patients of their follow-up appointments, the success of which is yet to be evaluated. The importance of follow-up controls needs to be discussed with all patients to improve adherence to their surveillance schedule. 

